# From failure to success: endoscopic management of difficult pancreatic cannulation after ampullary adenoma resection

**DOI:** 10.1055/a-2709-7289

**Published:** 2025-11-14

**Authors:** Shanbin Wu, Ying Yang, Xiujuan Zhang, Guodong Li, Haiyan Dong, Wei Li

**Affiliations:** 1 66310 Department of Gastroentroenterology, The First Affiliated Hospital of Shandong First Medical University and Shandong Provincial Qianfoshan Hospital, Jinan, China; 2 66310 Department of Pediatrics, The First Affiliated Hospital of Shandong First Medical University and Shandong Provincial Qianfoshan Hospital, Jinan, China

Endoscopic papillectomy is a standard treatment for ampullary adenomas, especially those with high-grade dysplasia 1 2 . Prophylactic pancreatic stenting is recommended to prevent postprocedural pancreatitis 1 2 . However, achieving deep pancreatic duct cannulation through the major papilla may be technically challenging, particularly after ampullectomy due to mucosal bleeding or anatomic variations such as pancreas divisum.

This report demonstrates a rescue approach using needle-knife fistulotomy at the minor papilla to overcome failed pancreatic access via the major papilla after endoscopic ampullectomy ( Video 1 ). A 59-year-old woman presented with a duodenal ampullary adenoma with high-grade intraepithelial neoplasia. Preoperative MRCP showed no evidence of ductal invasion ( Fig. 1 ). Endoscopic papillectomy was performed using a snare without submucosal injection, and hemostasis was achieved with coagulation forceps. Biliary cannulation was successful, but repeated pancreatic cannulation attempts failed, as the guidewire consistently exited through the minor papilla and could not reach the pancreatic tail, suggesting the possibility of incomplete pancreas divisum. Following biliary stenting, an ultra-precise needle-knife incision was performed at the minor papilla over the guidewire under direct endoscopic visualization. The guidewire was advanced into the pancreatic body and tail, enabling successful pancreatic stent placement.

Guidewire-assisted minor papilla fistulotomy enabled successful pancreatic stenting after failed major papilla cannulation post-ampullectomy.Video 1

**Fig. 1:**
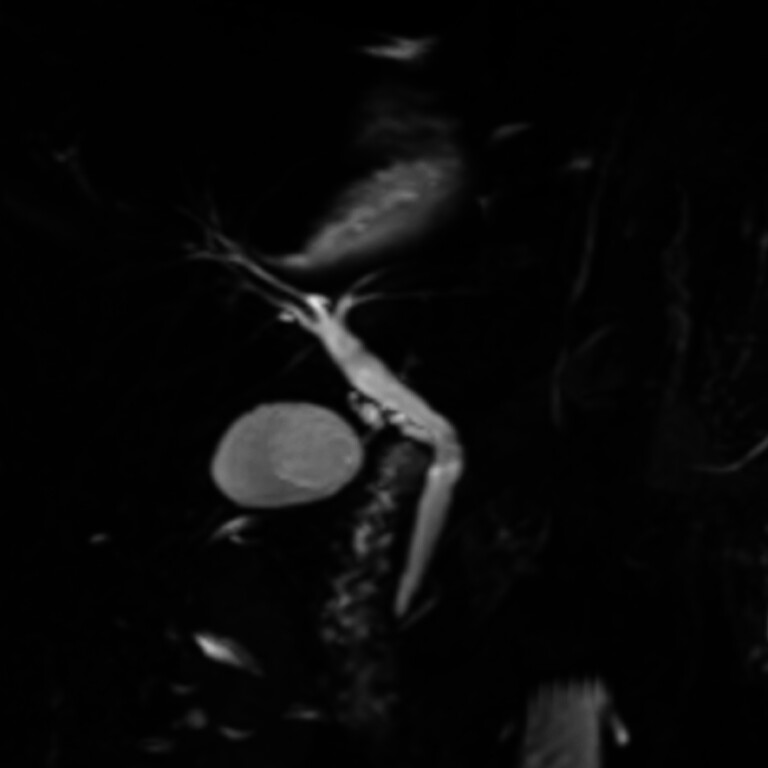
Preoperative MRCP showing an intact bile and pancreatic duct without evidence of ductal invasion.

This case highlights the utility of minor papilla needle-knife fistulotomy as a salvage technique for deep pancreatic access when major papilla cannulation fails. Guidewire-assisted minor papilla access provides real-time orientation, minimizes blind manipulation, and may reduce the risk of post-ERCP pancreatitis. It offers a technically feasible and safe strategy for pancreatic stenting in anatomically altered or complex cases following ampullectomy.

Endoscopy_UCTN_Code_TTT_1AR_2AD

## Notes

Endoscopy E-Videos https://eref.thieme.de/e-videos
*E-Videos* is an open access online section of the journal *Endoscopy* , reporting on interesting cases and new techniques in gastroenterological endoscopy. All papers include a high-quality video and are published with a Creative Commons CC-BY license. Endoscopy E-Videos qualify for HINARI discounts and waivers and eligibility is automatically checked during the submission process. We grant 100% waivers to articles whose corresponding authors are based in Group A countries and 50% waivers to those who are based in Group B countries as classified by Research4Life (see: https://www.research4life.org/access/eligibility/ ). This section has its own submission website at https://mc.manuscriptcentral.com/e-videos .
